# Intimate Partner Violence and Sexual Risk-Taking: Attachment Avoidance as a Linking Mechanism

**DOI:** 10.3390/bs15020239

**Published:** 2025-02-19

**Authors:** Jacqueline Woerner, Catalina Kopetz, Ximena Arriaga

**Affiliations:** 1 Department of Sociology, University of Central Florida, Orlando, FL 32816, USA; jacqueline.woerner@ucf.edu; 2 Department of Psychology, University of Central Florida, Orlando, FL 32816, USA; 3 Department of Psychology, Wayne State University, Detroit, MI 48202, USA; catalina.kopetz@wayne.edu; 4 Department of Psychological Sciences, Purdue University, West Lafayette, IN 47907, USA

**Keywords:** risky sex, casual sex, intimate partner violence, attachment avoidance

## Abstract

Why might women who experience intimate partner violence (IPV) become more likely to engage in risky sexual behavior? Women’s interest in casual sex may satisfy relational expectations and connection needs, while avoiding the types of close attachments that previously were violent. Specifically, attachment avoidance was tested as a mechanism linking IPV victimization and risky sexual behavior. Women who experienced (vs. did not experience) partner violence reported higher rates of risky sexual behavior, and this association was mediated by attachment avoidance (Study 1, *N* = 312; age range 18–58 years, *M* = 28). Making IPV salient via an experimental manipulation caused more avoidant perceptions (Study 2, *N* = 140; age range 19–57 years, *M* = 31), and inducing an avoidant mindset via an experimental manipulation caused greater sexual interest (Study 3, *N* = 128; age range 19–66 years, *M* = 33). These findings suggest that IPV disrupts expectations of security and reinforces a manner of connecting with partners that leads to risky sexual encounters.

## 1. Introduction

Sexual, psychological, and physical forms of intimate partner violence (IPV) in adult romantic involvements disproportionately affect women worldwide (Bacchus et al., 2018; Tjaden & Thoennes, 2000). Women who experience such IPV are more likely than men to be injured (Archer, 2000), experience fear (Cercone et al., 2005), and exhibit a strong physiological response to aggression (H. K. Kim et al., 2015). The negative outcomes of IPV are numerous, including mental health problems, loneliness and social isolation, difficulty forming and maintaining stable relationships, and increased susceptibility to physical illness (Bacchus et al., 2018; Dokkedahl et al., 2022; Stubbs & Szoeke, 2022; World Health Organization, 2021). Research also reveals that experiencing IPV is robustly associated with engaging in risky sexual behavior—including sex with multiple partners, casual sex, and inconsistent use of condoms (Brener et al., 1999; Campbell et al., 2004; Golder & Logan, 2011; Hamburger et al., 2004; Lang et al., 2011; Mittal et al., 2011)—which creates risk for contracting sexually transmitted infections.

Although an association of IPV with risky sexual behavior has been established, the underlying mechanisms that explain this association are poorly understood and their implications for interpersonal dynamics rarely studied. Very little is known about the relationship orientations that explain why women who have experienced IPV are likely to subsequently engage in risky sexual behavior. This research attempts to advance an explanation for the relationship between IPV and engagement in risky sexual behavior.

We suggest that women who previously experienced IPV will seek casual sex as a way of avoiding close emotional connections that previously were violent. Thus, a psychological state of avoidance may account for the link between IPV victimization and risky sexual behavior among women. Women, more so than men, are socialized to value emotional bonds characterized by intimacy and communion (Eagly et al., 2000). When relationships instead turn violent and become a source of pain, distress, and negative emotions, women may revise their relational expectations to become less trustful of relational intimacy—that is, less favoring of emotional bonds based on closeness and mutual reliance—and increasingly adopt thoughts and feelings that reflect a psychological state of attachment avoidance (Mikulincer & Shaver, 2016). We posit that women who experience IPV will become more avoidant of intimacy and more drawn to the detached nature of casual sexual encounters that characterize risky sexual behavior.

### 1.1. Intimate Partner Violence and Risky Sexual Behavior

The association between IPV and risky sexual behavior is commonly explained as reflecting a self-regulatory failure caused by stress or emotion dysregulation. Indeed, extensive research suggests that previous experience with IPV may increase sexually risky behavior through post-traumatic stress disorder (PTSD), depression (Cavanaugh et al., 2010; Smith et al., 2006), and the use of alcohol and drugs for self-medication (e.g., Deliramich & Gray, 2008; Littleton et al., 2007). This would suggest that victims of IPV engage in risky sexual behavior as a means of reducing the negative affective states caused by IPV (Walsh et al., 2012).

A different line of research suggests that sexual behavior, even in its risky forms, is an attempt to fulfill a need for interpersonal connection (Beaulieu et al., 2022; Birnbaum & Reis, 2019; Kopetz et al., 2014; Woerner et al., 2016). From this perspective, negative experiences in meaningful relationships (e.g., being emotionally hurt, bullied, or physically harmed) may predispose women to avoid emotional involvement and commitment and may increase their tendencies to engage in risky sexual behavior. Although research has documented a link between attachment insecurity and risky sexual behavior (see H. M. Kim & Miller, 2020 for a metanalysis), it is not clear why the association exists and what its implications are for understanding the relationship between IPV and risky sexual behavior. We suggest that rather than simply cut off all ties with others, women who experienced IPV may opt for casual sex, sex with multiple partners, and other forms of non-committed sexual behavior as an alternative means to fulfill one’s connection and sexual needs (Beaulieu et al., 2022) while avoiding the risk of violence from a relationship partner.

### 1.2. The Mediating Role of Attachment Avoidance

Previous research has suggested that insecurely attached individuals are at greater risk of IPV (Lafontaine et al., 2018; Slotter et al., 2020; Stefania et al., 2023). Here, we examine the opposite direction of this association to suggest that IPV experiences may revise relational expectations and specifically reinforce greater attachment avoidance. Research has documented the malleability of attachment orientations based on new experiences in relationships (see Arriaga et al., 2014, for increased security across time and Davila et al., 1999, for decreased security across time).

From a psychological perspective, people experience the greatest benefits of relational bonds when close others have been trustworthy, caring, loving, and reliably available for support when needed (B. C. Feeney & Collins, 2015; Fraley et al., 2013; Mikulincer & Shaver, 2016). These experiences reinforce stable expectations reflected by a secure attachment orientation, which then guides how people interact with others in the future. However, a person’s attachment orientation is not immutable; new experiences can revise attachment-relevant expectations and beliefs, which can alter a person’s attachment orientation in their romantic involvements (Arriaga & Kumashiro, 2019).

IPV violates relational expectations; the person who should be loving and caring instead becomes a source of emotional pain and, at times, physical harm (Arriaga et al., 2018). Being repeatedly hurt by close others to the point that one learns not to rely on others is specifically reflected in an adult avoidantly attached orientation, whereby a person has learned to be wary of closeness and intimacy (Mikulincer & Shaver, 2016). Painful experiences with others become salient in the mental representations (i.e., mental models) of relationship partners that manifest as avoidance. IPV victimization is precisely the type of experience that might reinforce attachment avoidant thoughts and feelings.

Chronically avoidant individuals are no less inclined than secure individuals to desire social connection with others (Carvallo & Gabriel, 2006; MacDonald & Borsook, 2010), but the means of satisfying that desire to connect with others is to remain relatively aloof and detached (Mikulincer & Shaver, 2016). Avoidant individuals typically do not seek emotional closeness in their sexual encounters (Beaulieu et al., 2022; Little et al., 2010). Indeed, avoidant individuals have a greater acceptance of casual sexual involvements, one-night stands, sex outside of established relationships, and sexual risk-taking (Davis et al., 2004; J. A. Feeney et al., 2000).

Sexual abuse (e.g., coercing sexual behavior), psychological abuse (e.g., belittling, controlling, and intimidating), and physical abuse (e.g., pushing), as reflected in IPV victimization, are all experienced as deeply painful (Arriaga et al., 2018). This research suggests that such painful moments in intimate relationships will reinforce avoidant thoughts and feelings; casual sex with no expectation of establishing an ongoing relationship satisfies connection needs while avoiding the dangers of past violent relationships (Kopetz & Orehek, 2015). Thus, IPV victims may become more likely to engage in form of risky sexual behavior characterized by low emotional involvement, not necessarily (or solely) because they seek to escape their stress and negative affect, but rather to satisfy connection needs (Kopetz et al., 2014; Woerner et al., 2016). As described above, female gender roles emphasizing intimacy and communion (Eagly et al., 2000; Wood et al., 1997) make women particularly susceptible to feeling undervalued when their partner’s behavior violates relational expectations (Ayduk et al., 1999).

### 1.3. Current Research

**Figure 1:**
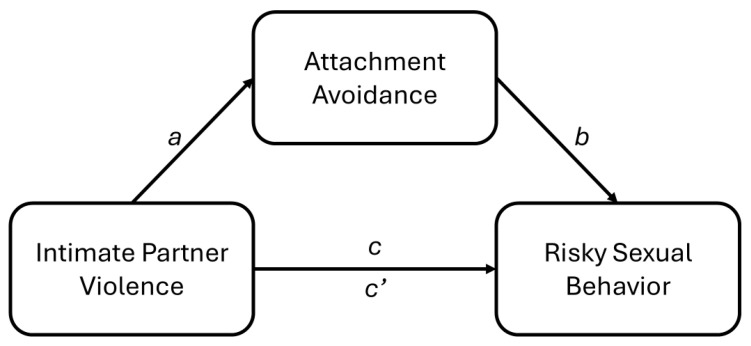
Conceptual model. Study 1 (cross-sectional) examined the indirect effect (*ab*) of IPV on risky sexual behavior through attachment avoidance as a mediating mechanism. Study 2 (experimental) examined the effect of IPV salience on attachment avoidance accessibility (*a*). Study 3 (experimental) examined the effect of an attachment avoidance manipulation on casual sex interest (*b*).

The aim of this research was to test whether women who experience IPV victimization adopt an avoidantly attached mindset with respect to close bonds, and in turn, whether attachment avoidance is associated with greater sexual interest in casual sex (i.e., sex with someone who is not a stable relational partner). This reasoning suggests that attachment avoidance will mediate the association of IPV victimization with casual sex. The conceptual model is presented in Figure 1.

Three studies tested the ideas. The studies assessed either self-reports of risky sexual behavior (e.g., multiple partners and condom use) or in-the-moment sexual interest in a non-intimate relationship (i.e., casual sex). Casual sex is not inherently risky. However, previous research shows that casual sex can pose risks as it relates to infrequent condom use (Bailey et al., 2008) and predicts higher rates of STIs (Ann Lyons, 2017). IPV victimization was assessed with a measure that examines sexual, psychological, and physical IPV.

Study 1 (correlational) assessed (a) the association of previous IPV victimization experiences with engagement in risky sexual behavior and (b) attachment avoidance as a mediator of the IPV-risky sexual behavior association, as depicted in Figure 1. Studies 2 and 3 specifically tested the causal role of attachment avoidance in accounting for the association of IPV victimization with casual sex. These two studies combined used the strongest test possible of a mediating causal process (see MacKinnon & Pirlott, 2015, on causal inference in mediation models): Study 2 experimentally varied the salience of IPV experiences (independent variable) to see if it causes more avoidant thoughts and feelings (mediator) and Study 3 experimentally varied an attachment-avoidant mindset (mediator) to see if it causes an inclination to pursue casual sex (dependent variable).

### 1.4. Ethical Issues

All studies were advertised to potential participants as a study on social, dating, and sexual behaviors. Potential participants were informed that they would be asked questions about past sexual and dating experiences; were instructed they could skip questions that made them uncomfortable answering while remaining in the study; were informed they could withdraw at any point without penalty; and were also provided with information about mental health hotlines and referral resources in the even that they experienced any distress reporting on past experiences. For Studies 2 and 3 with an experimental manipulation, participants were fully debriefed of the study purpose upon completing they study tasks. All procedures were approved by an Institutional Review Board, and responses were anonymous and confidential.

### 1.5. Data Preparation and Statistical Software

Prior to analysis, all data were reviewed for quality. Attention checks were embedded in each survey, and data were excluded for failed attention checks, duplicate response IDs, and not completing the survey past the first measure. Regression assumptions (e.g., normality of residuals) were checked prior to conducting relevant analyses. All analyses were conducted with SPSS version 29.

## 2. Study 1

### 2.1. Introduction

Study 1 was based on a cross-sectional survey that aimed to test whether an avoidant attachment orientation mediates the association of women’s IPV sexual, psychological, and/or physical victimization experiences with casual sexual behavior. The hypothesis was that having IPV victimization experiences (versus not) would be associated with higher engagement in risky sexual behavior, and this association would be mediated by higher attachment avoidance.

### 2.2. Method

#### 2.2.1. Participants and Procedure

The sample was 312 women who were recruited through Mechanical Turk to complete an online study administered via Qualtrics. Participants were required to be heterosexual and unmarried, and their age ranged from 18 to 58 years (*M* = 27.63, *SD* = 5.26). Self-reported race/ethnicity was 71.1% (*n* = 221) Caucasian/White; 9.3% (*n* = 29) African American/Black; 6.4% (*n* = 20) Multiracial; 6.1% (*n* = 19) Asian, East Asian, or Pacific Islander; 5.8% (*n* = 18) Hispanic; 0.3% (*n* = 1) Arabic or Middle Easterner; 0.3% (*n* = 1) Native American/American Indian; and 0.9% (*n* = 3) another identity. Annual household income ranged from less than USD 10,000 to more than USD 75,000 with a median of USD 30,000–39,999 and 45.5% (*n* = 142) completed a Bachelor’s degree or higher. In total, 41% (*n* = 128) of participants reported being single or not dating exclusively, 28.8% (*n* = 90) reported being in an exclusive dating relationship, 28.5% (*n* = 89) were living with a romantic partner, and 1.6% (*n* = 5) were divorced or widowed.

Participants completed a self-administered online survey that guided them through an initial consent process and then completion of the measures described below. The measures were presented in the order they appear below.

#### 2.2.2. Measures

- Past IPV victimization

Lifetime IPV victimization was assessed with the Conflict Tactics Scale II—Short Form (CTS2; Straus & Douglas, 2004). Participants were presented with six partner acts and indicated how many times each had occurred, ranging from 0 *never happened* to 6 *happened more than 20 times*. Two items tapped sexual, psychological, and physical victimization, respectively, one assessing mild and the other assessing severe behaviors. Consistent with recommendations from past research for scoring IPV prevalence with the CTS2 (Straus & Douglas, 2004), variables were coded as follows: 0 = the absence of IPV, 1 = the presence of each type of IPV. Thus, three variables were created reflecting a lifetime history for each of three types of IPV: (1) sexual IPV, (2) psychological IPV, or (3) physical IPV. Additionally, a combined past IPV victimization score was computed by summing the three variables for each IPV type, with scores ranging from 0 *never experienced any type of IPV* to 3 *experienced sexual, psychological, and physical IPV*. Higher scores indicated more past types (i.e., sexual, psychological, and physical) of IPV victimization.

- Risky sexual behavior

Engagement in risky sexual behavior over the past year was assessed with five items used in past research with young adults (Cooper, 2010). Items 1 and 2 assessed the total number of past year sexual partners and their number of past year casual partners (0 *zero*, 1 *one*, 2 *two*, 3 *three to five*, 4 *six to ten*, or 5 *more than 10 partners*). Item 3 assessed condom use frequency with casual partners, with the following response options: 0 *no casual partners*, 1 *every time*, 2 *often*, 3 *sometimes*, 4 *rarely*, and 5 *never*. Item 4 assessed frequency of drug and alcohol use prior to sexual activity (0 *do not ever consume drugs or alcohol*, 1 *never*, 2 *rarely*, 3 *sometimes*, 4 *often*, and 5 *every time*). In item 5, participants endorsed (checked) whether or not they discussed each of five sex-related topics with their most recent sexual partner prior to engaging in sexual activity (pregnancy, sexually transmitted infections, partner’s sexual history, IV drug use history, and use of condoms or birth control); responses were summed and reverse coded so that a higher count for item 5 indicated *less* discussion of risks, with scores ranging from 0 *discussed all risks* to 5 *didn’t discuss any risks*. All five items had responses that ranged from 0 to 5 and were averaged (α = 0.58) such that higher scores indicated riskier sexual behavior.

- Attachment avoidance

Attachment avoidance was assessed with the 36-item Experiences in Close Relationship Scale (ECR; Brennan et al., 1998), which measures attachment avoidance and attachment anxiety. Eighteen items assessed attachment avoidance, reflecting the extent to which participants are uncomfortable with emotional closeness (e.g., “I get uncomfortable when a romantic partner wants to be very close”; “I find it difficult to allow myself to depend on romantic partners”), with response options ranging from 1 *strongly disagree* to 5 *strongly agree*. Responses were averaged (α = 0.94) such that higher scores reflected greater attachment avoidance. An additional 18 items assessed attachment anxiety (α = 0.93) and responses were averaged such that higher scores reflected greater attachment anxiety.

### 2.3. Results and Synthesis

#### 2.3.1. Descriptive Results and Simple Correlations

A majority reported at least one past instance of IPV victimization; 29.2% (*n* = 91) of women experienced past sexual IPV, 72.4% (*n* = 226) experienced past psychological IPV (mild and severe combined; 20.3% past severe psychological IPV), and 26.0% (*n* = 81) experienced past physical IPV. Combining across sexual, psychological, and physical types of IPV revealed that 24.8% of women had never experienced any type of IPV, 36.9% experienced one type, 23.5% experienced two types, and 14.7% experienced all three types.

Prior to testing the mediation hypothesis, bivariate correlations were examined. Results indicated that past experiences with each type of IPV were associated with risky sexual behavior in the past year: sexual IPV (*r* = 0.28, *p* < 0.001), psychological IPV (*r* = 0.13, *p* = 0.025), and physical IPV (*r* = 0.24, *p* < 0.001). Furthermore, risky sexual behavior was positively correlated with the number of types of IPV (r = 0.28, *p* < 0.001).

#### 2.3.2. Analysis Strategy to Assess Mediation

Analyses were conducted in SPSS version 29 with the PROCESS macro v4.2 (Hayes, 2017). To test the mediation hypothesis, indirect effects analyses were conducted via four separate models for each IPV variable (the presence vs. absence of sexual, psychological, and physical IPV) and the total count of IPV types (0 through 3) as the independent variable, attachment avoidance as the mediator, and risky sexual behavior as the outcome.

Specifically, each model tested the following: (1) whether each IPV variable is associated with attachment avoidance (*a*); (2) whether attachment avoidance is associated with risky sexual behavior (*b*); and (3) the indirect effect of each IPV variable on risky sexual behavior through avoidant attachment (*ab*).

Evidence of mediation is established through statistical significance of the indirect effect (*ab*) (e.g., see Hayes & Rockwood, 2017). We also examined (4) the direct effect of IPV on risky sexual behavior (*c*’) and (5) the total effect of IPV on risky sexual behavior (*c*), such that *c* = *c*′ + *ab*.

All models controlled for attachment anxiety, another dimension of insecurity, to isolate the association of attachment avoidance with other variables.

#### 2.3.3. Test of the Hypothesized Mediation Model

The first model examined sexual IPV. Sexual IPV was associated with higher attachment avoidance (*b* = 0.22, *SE* = 0.10, *p* = 0.021 [*a*]), and attachment avoidance was associated with higher rates of risky sexual behavior (*b* = 0.23, *SE* = 0.06, *p* < 0.001 [*b*]), *R*^2^ = 0.15. The indirect effect (*ab*) of sexual IPV on avoidant attachment was significant, as evidenced by confidence intervals that did not contain zero (*b* = 0.05, *SE* = 0.03, 95% bootstrapped 95% CI [0.01, 0.10]). The direct (*c*’) and total (*c*) effects of sexual IPV were statistically significant (*p* < 0.001).

The second model examined psychological IPV. Results indicated that psychological IPV was not significantly associated with avoidance (*b* = 0.03, *SE* = 0.10, *p* = 0.801 [*a*]); therefore, the indirect effect (*ab*) was not significant (*b* = 0.01, *SE* = 0.02, 95% bootstrapped CI [−0.04, 0.06]). However, because the majority of the sample (72.4%) endorsed psychological IPV, we conducted a follow-up analysis with just severe psychological IPV as the independent variable, which had lower rates (20.3%) that were more comparable to rates of sexual and physical IPV in the sample. The results of this indirect effects analysis showed that severe psychological IPV was significantly associated with greater attachment avoidance (*b* = 0.41, *SE* = 0.11, *p* <0.001 [*a*]), and attachment avoidance was associated with greater risky sexual behavior (*b* = 0.19, *SE* = 0.06, *p* = 0.001 [*b*]), *R*^2^ = 0.13. The indirect effect (*ab*) of severe psychological IPV on risky sexual behavior via attachment avoidance was significant (*b* = 0.08, *SE* = 0.03, 95% bootstrapped CI [0.02, 0.15]). The direct (*c*’) and total (*c*) effects of severe psychological IPV were statistically significant (*p* < 0.001).

The third model examined physical IPV. The results showed that physical IPV was associated with higher attachment avoidance (*b* = 0.29, *SE* = 0.10, *p* = 0.005 [*a*]), and attachment avoidance was associated with increased risky sexual behavior (*b* = 0.22, *SE* = 0.06, *p* < 0.001 [*b*]), *R*^2^ = 0.12. The indirect effect (*ab*) was significant (*B* = 0.06, *SE* = 0.03, 95% CI [0.01, 0.13]). The direct (*c*’) and total (*c*) effects of physical IPV were also statistically significant (*p* < 0.001).

The final model examined the effect of the count of types of IPV (i.e., sexual, psychological, and physical) combined, with cumulative IPV as the independent variable, attachment avoidance as the mediator, and risky sexual behavior as the outcome. IPV victimization (combined) was associated higher attachment avoidance (*b* = 0.11, *SE* = 0.05, *p* < 0.020 [*a*], and attachment avoidance was significantly associated higher rates of risky sexual behavior (*b* = 0.22, *SE* = 0.06, *p* < 0.001 [*b*]), *R*^2^ = 0.14; the indirect effect (*ab*) was significant (*b* = 0.02, *SE* = 0.01, 95% CI [0.003, 0.05]). The direct (*c*’) and total (*c*) effects of IPV were also significant (*p* < 0.001).

Full model results (including unstandardized coefficients, standard errors, and 95% confidence intervals for each effect) are shown in Table 1.

Finally, to test whether the mediating mechanism is specific to attachment avoidance, all models were rerun with attachment anxiety as the mediator (controlling for avoidant attachment); none of the indirect effects of attachment anxiety were statistically significant.

#### 2.3.4. Synthesis

The Study 1 results supported the idea that attachment avoidance mediates the link between IPV victimization and risky sexual behavior. IPV victimization was associated with risky sexual behavior. IPV victimization also was associated attachment avoidance, which in turn was associated with risky sexual behavior. The mediation test yielded support for attachment avoidance specifically and was not significant when testing attachment anxiety as the mediator.

Study 1 replicated past research showing a correlation of IPV victimization and risky sexual behavior (Lang et al., 2011; Littleton et al., 2007). Study 1 further demonstrated that victims of IPV may pursue risky sexual behavior because it affords an opportunity for interpersonal connection without the emotional commitments that have been hurtful in the past.

Study 1 provides the initial proof of concept for the mediation model proposed in this research, which suggest that attachment avoidance is a causal mechanism linking IPV victimization to risky sexual behavior. However, Study 1 was limited by correlational data, which do not afford evidence of causal associations.

## 3. Study 2

### 3.1. Introduction

Studies 2 and 3 were designed as experiments that can provide greater causal inference on the mediating role of attachment avoidance in accounting for the IPV victimization link with casual sex (see MacKinnon & Pirlott, 2015, on causal inference in mediation models).

Study 2 varied the independent variable of IPV victimization by experimentally manipulating mental accessibility of past IPV experiences to assess its effect on avoidant thoughts and feelings. It was hypothesized that making past IPV experiences mentally accessible through experimental manipulation (versus not) will cause a person to recall more current relationships that reflect avoidance.

### 3.2. Method

#### 3.2.1. Participants and Procedure

The sample was 140 women who were recruited through Mechanical Turk to complete an online study administered via Qualtrics. The current analysis was based on participants who reported having engaged in sexual activity with a man. The online materials manipulated (between subjects) the salience of a previous IPV-related experiences and assessed attachment avoidance. A power analysis using G*Power determined that a useable sample of *N* = 128 (64 participants in each of two conditions) would be sufficient to detect a medium effect size (based on bivariate associations in Study 1) with 0.80 power and an alpha of 0.05.

Participants’ age ranged from 19–57 (*M* = 30.94, *SD* = 8.15 and self-reported their race/ethnicity as follows: 72.9% (*n* = 102) Caucasian/White; 6.4% (*n* = 9) African American/Black; 5.0% (*n* = 7) Hispanic; 2.9% (*n* = 4) Asian, East Asian, or Pacific Islander; 2.1% (*n* = 3) Multiracial; 1.4% (*n* = 2) Native American/American Indian; and 9.3% (*n* = 13) another identity or did not respond. Additionally, 17.1% of participants were single, 13.6% were in a dating relationship, 17.1% were living with a romantic partner, 39.3% were married, 2.9% were divorced, and 0.7% were widowed, and data were missing from 9.3%. Participants’ annual household income ranged from less than USD 10,000 to more than USD 100,000 (*Md* = USD 40,000–49,999), and 51.4% (*n* = 72) had attained a bachelor’s degree or higher.

Participants completed a self-administered survey that guided them through an initial consent process and then the experimental tasks. The survey included a recall task that introduced the experimental manipulation, followed by measures in the order they appear below.

#### 3.2.2. Manipulated and Measured Variables (Presented in the Order They Appear Below)

- Manipulation of intimate partner violence salience

The salience of past IPV experiences was manipulated through a recall task. Participants were instructed to recall a specific event in their past, whereby the recall instructions varied in the experimental vs. control condition, and then were directed to close their eyes, picture the recalled experience, and recall how they felt at the time.

In the experimental condition, participants were asked to recall a negative experience they had with a romantic or sexual partner. Participants were asked to think about a time when their partner had been aggressive or violent towards them; if nothing like this had happened, participants were instructed to think about the worst argument they had experienced with a relationship partner, or to recall when they had felt scared by something a partner said or did.

In the control condition, participants were instead asked to recall an experience when they did not finish a school or work assignment on time; if nothing like this had happened, participants were instructed to think about a time when they submitted their lowest quality work, or a time they felt rushed to complete an assignment.

Similar manipulations have been successfully employed to manipulate mortality salience (Menaker & Miller, 2013; Rosenblatt et al., 1989) and thoughts about rape (Bohner & Schwarz, 1996; Bohner et al., 1993). To ensure that participants were paying attention, they were later asked to describe the topic that they were previously asked to reflect upon. All participants successfully completed this task.

- Past IPV experiences

Participants’ lifetime IPV experiences were assessed with the same measure used in Study 1 (CTS2-Short Form; Straus & Douglas, 2004).

- Own current attachment orientation

Attachment tendency was assessed with a measure that requires participants to read three attachment orientation descriptions and select the one that best characterizes the way they generally feel in close relationships (Hazan & Shaver, 1987). Each paragraph describes an attachment orientation: secure: “I find it relatively easy to get close to others and am comfortable depending on them and having them depend on me. I don’t often worry about being abandoned or about someone getting too close to me”; avoidant: “I am somewhat uncomfortable being close to others. I find it difficult to trust them completely, difficult to allow myself to depend on them. I am nervous when anyone gets too close, and often, love partners want me to be more intimate than I feel comfortable being”; and anxious: “I find that others are reluctant to get as close as I would like. I often worry that my partner doesn’t really love me or won’t want to stay with me. I want to merge completely with another person, and this desire sometimes scares people away.” A categorical variable was created to reflect the participant’s general attachment tendency.

- Attachment avoidance accessibility in recalled relationships

After indicating their current attachment orientation, participants completed a task to assess the mental accessibility of current relationships that reflect each attachment orientation (secure, avoidant, and anxious). Specifically, participants first recalled and listed the names/initials of five close others and the type of relationship with each person (e.g., dating partner and parent). Participants then assigned an attachment style that characterized their bond with person they listed (each name/initial); using the descriptions above of a secure style vs. avoidant style vs. anxious style, they indicated which style characterized each relationship. Scores were computed to represent the proportion of recalled (listed) relationships that were a secure style, avoidant style, or anxious style. The proportion of recalled relationships that fit an avoidance style (versus a secure style or anxious style) was used to indicate attachment avoidance accessibility.

### 3.3. Results and Synthesis

#### 3.3.1. Descriptive Results

As in Study 1, a majority reported past IPV victimization: 81.4% of the women experienced past psychological IPV, 34.3% past physical abuse, and 32.9% past sexual IPV. Furthermore, 11.4% experienced no past IPV, 39.4% experienced one type (i.e., sexual, psychological, or physical), 29.5% experienced two types, and 19.7% experienced all three types.

#### 3.3.2. Hypothesis Tests

**Figure 2:**
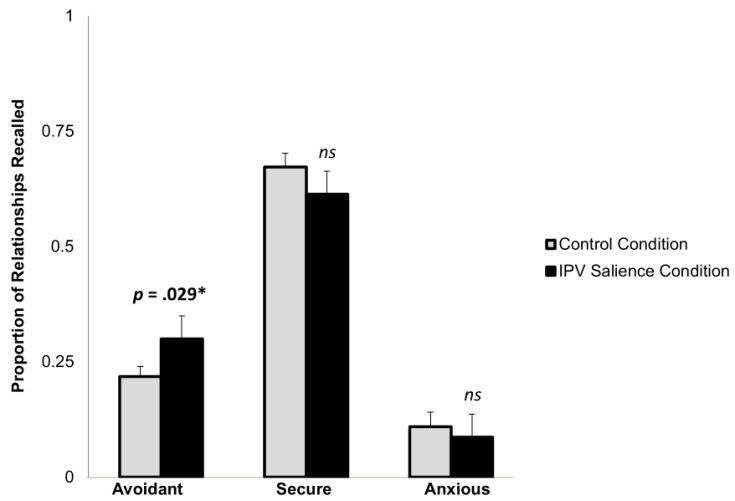
Study 2; Effect of IPV salience on attachment accessibility. * *p* < 0.05.

Participants in the IPV salience condition were no more likely than those in the control condition to describe their own current attachment orientation as reflecting avoidant style, as indicated by a chi-square analysis, *χ*^2^(2) = 3.04, *p* = 0.219. However, as hypothesized and shown in Figure 2, an ANOVA indicated that participants in the IPV salience condition recalled a greater proportion of avoidant relationships (*M* = 0.30 [30%], *SD* = 0.25), compared to participants in the control condition (*M* = 0.22 [22%], *SD* = 0.19), *F*(1,138) = 4.86, *p* = 0.029. A second ANOVA revealed this effect was not significant when controlling for their past IPV experiences, *F*(1,129) = 3.13, *p* = 0.079. Importantly, a third ANOVA revealed that the IPV salience condition did not affect the proportion of relationship recalled that fit a secure style, *F*(1,138) = 1.66, *p* = 0.200, or anxious style *F*(1,138) = 0.64, *p* = 0.426.

#### 3.3.3. Synthesis

The results showed that making past IPV experiences mentally accessible and salient facilitated the recall of one’s avoidant relationships. The IPV salience manipulation did not significantly affect one’s stable attachment orientation, but it affected the accessibility of relationships that uniquely have avoidant features, rather than anxious or secure features. These results suggest that making past IPV experiences salient leads to perceiving current relationships through an avoidant mindset, which can protect against the type of closeness that previously led to harmful experiences.

Study 2 provided evidence supporting a causal relationship between recalling past IPV victimization experiences and avoidant thoughts and feelings. The limitation of Study 2 is that it did not test a causal association between attachment avoidance and interest in casual sex, which was the focus of Study 3.

## 4. Study 3

### 4.1. Introduction

Study 3 included a manipulation to induce an avoidant mindset. This approach of experimentally manipulating the presumed mediating variable provides greater causal evidence (MacKinnon & Pirlott, 2015) of the role of attachment avoidance in accounting for the IPV victimization association with casual sex.

To induce an avoidant mindset, heterosexual women: (1) read a vignette in which they imagined a first date with a man that either induced an avoidantly attached mindset (experimental condition) or securely attached mindset (control condition) and (2) recalled a past experience that represented the same attachment mindset that had been induced in step 1. Participants then continued the vignette; after the initial date, they are in a new setting where they meet a new man and rate their sexual interest. This study did not induce an attachment anxious mindset given the results from Studies 1 and 2 that specifically implicate attachment avoidance.

Study 3 tested the hypothesis that adopting an avoidantly attached mindset, as compared with a securely attached mindset, would cause greater sexual interest and greater sexual risk-taking intentions.

### 4.2. Method

#### 4.2.1. Participants and Procedure

The sample was 133 single, heterosexual women who were recruited through Mechanical Turk to complete an online study administered via Qualtrics. Single and heterosexual women were specifically recruited because the experimental task required imagining a first date with a man, which would be difficult for involved or non-heterosexual women. As described earlier, participants were excluded from analysis if they failed attention checks; furthermore, they were excluded if they indicated it was extremely difficult to imagine themselves in the situation, stopped the survey before completing the manipulation, reported that they were in a committed romantic relationship, or stated in the open-ended section that they had never had a similar experience. A power analysis using G*Power determined that a useable sample of *N* = 128 (64 participants in each of two conditions) would be sufficient to detect a medium effect size (based on findings from the previous studies) with 0.80 power and an alpha of 0.05.

In the sample of *N* = 133 participants, *n* = 70 were randomly assigned to the secure control condition and *n* = 63 were randomly assigned to the experimental avoidant condition. Participants were between 19 and 66 years of age (*M* = 33.43, *SD* = 9.67), and self-reported their race/ethnicity as follows: 74.4% Caucasian/White; 15.0% African American/Black; 5.3% Asian, East Asian, or Pacific Islander; 4.5% Hispanic; and 0.8% Multiracial. Participants’ annual household income ranged from less than USD 10,000 to more than USD 100,000 (*Md* = USD 40,000–49,999) and 57.9% had attained a bachelor’s degree or higher.

Participants completed a self-administered survey that guided them through an initial consent process and then the experimental tasks. The survey included vignette and recall tasks that enacted the experimental manipulation, followed by measures in the order they appear below.

#### 4.2.2. Manipulated and Measured Variables (Presented in the Order They Appear Below)

- Manipulation of an attachment avoidance mindset

An avoidant mindset was induced with two tasks. Participants (1) read a vignette about going on a first date and then (2) recalled and described previous experiences that reinforced the manipulated mindset.

In the first task, participants read a vignette that guided them to imagine being on first date with “Michael”, who they met for dinner; Michael then engages in varying behaviors depending on the experimental condition.

In the secure (control) condition, Michael seems interested and engages in appropriate first date conversation. The scenario includes events that reflect feeling securely attached, based on Hazan and Shaver’s (1987) secure attachment description: “You find that it is relatively easy to get close to Michael and you feel comfortable depending on him. With Michael, you don’t worry about being abandoned by him and you don’t worry about him getting too close to you.”

In the avoidant (experimental) condition, Michael is annoying and asks invasive questions, and eventually, the person with Michael (whose perspective the participant is asked to adopt) wants to be doing other things and checks their phone. The experimental scenario includes events that reflect feeling avoidantly attached, based on Hazan and Shaver’s (1987) avoidant attachment description: “You are now thinking that you are somewhat uncomfortable being too close to Michael. You find it difficult to trust him completely and find it difficult to allow yourself to depend on him. With Michael, you feel yourself getting nervous when he tries to get too close to you and you feel he wants to be more intimate than you felt comfortable being.”

In the second task of the avoidant mindset manipulation, participants recalled and described an experience (or multiple experiences) matching the scenario above. This task aimed to connect the hypothetical scenario with actual past experiences.

Participants in the avoidant condition were instructed to: “Consider similar experiences you’ve had where you felt your partner was imposing too much on your relationship, and you felt the need to distance yourself. Please take a few minutes to reflect upon a time where you felt this way. Specifically, think about an experience (or multiple experiences) in which you felt a partner was being too intense or overbearing, or had unrealistic relationship expectations, such that you wanted to pull away. Experiences like this may involve a partner who demanded too much loyalty or investment from you, or a partner who seems volatile when they don’t feel sufficiently loved or valued.”

In the secure condition, participants were instructed to: “Consider similar experiences you’ve had where your partner and you wanted the same level of involvement, and you felt comfortable and secure in a relationship. Please take a few minutes to reflect upon a time where you felt this way. Specifically, think about an experience (or multiple experiences) in which you felt a partner was reasonable and easy to connect with, and you had similar expectations of each other, such that it was easy to get close to this person. Experiences like this may involve a partner who provided the right level of involvement and was not overly demanding or prone to being volatile, or insecure.”

The manipulation check assessing whether the avoidant mindset task had the intended effect required participants to answer 6 items tapping their attachment-relevant thoughts while imagining the interaction with Michael (2 items each assessing avoidant, anxious, and secure attachment; Brennan et al., 1998; Carnelley & Rowe, 2007); response options ranged from 1 *strongly disagree* to 5 *strongly agree.* Items were averaged to reflect avoidant (α = 0.84), anxious (α = 0.81), or secure (α = 0.94) thoughts and feelings, where higher scores reflected each attachment orientation.

**Figure 3:**
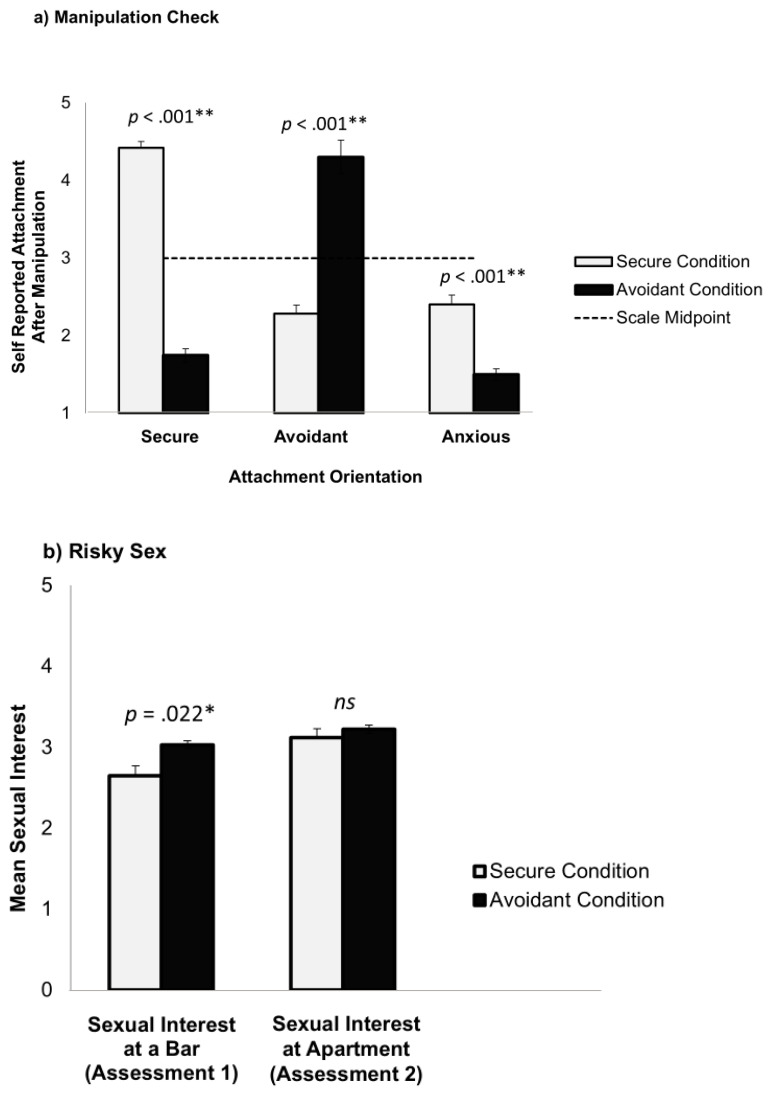
Study 3: Effect of attachment manipulation on (**a**) self-reported attachment and (**b**) sexual interest. * *p* < 0.05, ** *p* < 0.001.

The inducement of an avoidant mindset in the hypothetical date with Michael worked as intended (see Figure 3a). A series of ANOVA tests revealed that participants in the avoidant condition reacted with greater avoidance to the date with Michael (*M* = 4.30, *SD* = 0.77), relative to participants in the secure condition (*M* = 2.29, *SD* = 1.03), *F*(1,131) = 161.12, *p* < 0.001; participants in the secure condition reacted with greater security (*M* = 4.42, *SD* = 0.67), relative to participants in the avoidant condition (*M* = 1.75, *SD* = 0.87), *F*(1,131) = 402.45, *p* < 0.001; and participants also reported relatively low levels of attachment anxiety (i.e., lower than the midpoint of the scale) in both conditions, albeit higher in the secure condition (*M* = 2.40, *SD* = 1.09) than in the avoidant condition (*M* = 1.50, *SD* = 0.87), *F*(1,131) = 27.52, *p* < 0.001.

- Sexual interest and sexual risk-taking intention

Participants subsequently continued the hypothetical scenario task. They were told to imagine that after the date with Michael, they headed to a local bar to meet up with some close friends. Participants were asked to imagine that an attractive man at the bar approached them, and they engaged in conversation. His name was Adam, and as it was getting late, he asked, “Do you want to hang out and talk some more? My place is right down the street.”

To assess interest in having sex with Adam while at the bar (i.e., first assessment), participants responded to four questions using a 1–5 response scale: how interested they are in Adam, how much they would like to have sex with him, how much they want to go home with him, and how likely they’d be to have sex with him if they went home together. Responses for these four items were averaged, with higher scores indicating higher sexual interest (α = 0.78).

Participants then imagined that they left with Adam to go to his apartment, where they sat on the couch and began to kiss. The text leads participants to believe that they were both enjoying their interaction. To assess sexual interest in Adam’s apartment (i.e., second assessment), participants responded to an additional 4 items using a 1 to 5 response scale: their desire to have sex with Adam, interest in having sex if a condom wasn’t available, their interest in engaging in sexual activities not including intercourse, and how sexually aroused they were in the situation. Responses for these items were averaged, with higher scores indicating higher sexual risk-taking (α = 0.85).

- Past IPV experiences

Next, IPV (sexual, psychological, and physical IPV) was assessed to provide descriptive information about the sample. Participants’ lifetime IPV experiences were assessed with the same measure used in Studies 1 and 2 (CTS2, Straus & Douglas, 2004).

- Past risky sexual behavior

Next, past-year risky sexual behavior was assessed to include in analyses as a covariate. It was measured with a set of eight items assessing number of sexual partners, condom use with casual partners (consistent with the measure described in the previous studies), as well as behaviors relevant to the vignette that captured sexual behaviors characterized by low emotional investment, such as going to bars/parties with the intent of “hooking up” or having sex with someone. Responses were standardized due to different scaling and averaged such that higher scores indicate riskier behavior (α = 0.70).

### 4.3. Results and Synthesis

#### 4.3.1. Descriptive Results and Simple Correlations

As in Studies 1 and 2, a majority reported at least one past instance of IPV victimization; 46.6% of women experienced past sexual IPV, 80.3% experienced past psychological IPV (mild and severe combined), 20.5% past severe psychological IPV, and 30.5% past physical IPV. Past IPV victimization was not associated with sexual interest or sexual risk-taking with Adam (the hypothetical man at the bar). Importantly, sexual interest with Adam both assessments was significantly associated with participants’ past engagement in risky sexual behavior (*r* = 0.31, *p* < 0.001; *r* = 0.26, *p* = 0.022; respectively).

#### 4.3.2. Hypothesis Tests

ANOVAs were conducted to examine the effect of the attachment manipulation on sexual interest with hypothetical Adam, initially at bar (first assessment) and later at his apartment (second assessment). The results are shown in Figure 3b.

Participants in the avoidant mindset condition reported greater sexual interest with hypothetical Adam while at the bar (first assessment; *M* = 3.03, *SD* = 0.87), relative to secure mindset participants (*M* = 2.65, *SD* = 1.03), *F*(1,131) = 5.41, *p* = 0.022. This effect remained significant when controlling for past risky sexual behavior *F*(2,130) = 5.95, *p* = 0.044, suggesting that momentarily induced avoidant tendencies had a unique effect on participants’ sexual interest with someone they just met.

The effect of the manipulation on the intention to have sex with Adam while at his apartment (second assessment) was not significant (avoidant condition: *M* = 3.22, *SD* = 0.94; secure condition: *M* = 3.12, *SD* = 1.00), *F*(1,131) = 0.35, *p* = 0.554, and remained nonsignificant when controlling for past risky sex, *F*(2,130) = 0.37, *p* = 0.543.

#### 4.3.3. Synthesis

The Study 3 results were that experimentally inducing an avoidant (versus secure) attachment mindset increased interest in casual sex (at the bar; first assessment), but it did not increase following through with casual sex (at the apartment; second assessment). When tested with a hypothetical scenario, inducing an avoidant mindset may cause women to have greater interest in casual sex with a recently met person, but the impact may be limited to sexual interest and not affect the subsequent intention to engage in casual sex.

Why might the effect of an avoidant mindset have been limited in impact? First, measures of risky sexual behavior are difficult to assess (Leigh & Stall, 1993) and notoriously susceptible to social desirability bias. Participants might have deemed it socially inappropriate to report wanting to have sex with a stranger. Hypothetical scenarios lack the experimental realism of actual situations and may be more susceptible to socially desirable responses. Second, the avoidantly attached orientation that presumably causes risky sexual behavior is driven by powerful experiences that have shaped stable mental models. They may be partially malleable but require strong new experiences to shift in more lasting ways.

## 5. General Discussion

### 5.1. Summary of Results

Three studies provided support for the model depicted in Figure 1. In addition to replicating past findings, the current research directly tested a novel model using rigorous experimental methods for establishing mediation (MacKinnon & Pirlott, 2015). All studies relied on samples of adult women, who were the target population. The measure of IPV victimization assessed past sexual, psychological, and physical IPV. The measure of risky sexual behavior relied either on direct reports of risky sexual behavior or an assessment of interest in casual sex.

Study 1 provided correlational support for the association of IPV victimization with attachment avoidance among women, which in turn was associated with risky sexual behavior. Consistent with a mediation model, the indirect effect of IPV victimization on risky sexual behavior though attachment avoidance was significant, but the indirect effect was not significant when testing for attachment anxiety.

Studies 2 and 3 provided stronger evidence of avoidance as a causal mechanism mediating the association of IPV victimization with interest in casual sex. In Study 2, making past IPV experiences salient (versus non-IPV experiences) caused avoidant thoughts and feelings; in Study 3, an avoidant (versus secure) mindset caused interest in casual sex.

The current studies replicated previous research demonstrating correlations among: (a) IPV victimization and risky sexual behavior (direct replication; Lang et al., 2011; Littleton et al., 2007), (b) IPV victimization and attachment insecurity (conceptual replication focused on avoidance; Stefania et al., 2023), and (c) avoidance and sex involving lower levels of closeness (conceptual replication; Beaulieu et al., 2022). In addition to replicating past findings, the current research directly tested a mediation model using rigorous methods for establishing mediation (MacKinnon & Pirlott, 2015). The test of causality in Studies 2 and 3 yielded findings that were mostly consistent with Figure 1, with partial support for the effect of an avoidant mindset on casual sex, which was tested in a hypothetical scenario with a newly acquainted partner; the effect occurred for interest in casual sex but not for the intention to actually engage in casual sex.

### 5.2. Broader Implications

IPV victimization leads to several psychological and physical problems (Dokkedahl et al., 2022; Stubbs & Szoeke, 2022 for a review). Interestingly, an unexpected outcome for women who experience IPV victimization is an increased likelihood to engage in risky sexual behavior (Brener et al., 1999; Campbell et al., 2004; Golder & Logan, 2011; Hamburger et al., 2004; Lang et al., 2011; Mittal et al., 2011).

Why might women who experience IPV become more likely to engage in risky sex? A common explanation is that IPV victims engage in risky sexual behavior, alcohol and drug use, and other potentially harmful behavior to escape stress and negative affect (Walsh et al., 2012). While this explanation accounts for the use of short-term strategies to escape psychological pain, it does not account for the ways in which IPV experiences revise their enduring mental representations and expectations about relational bonds. Women may be particularly affected given societal pressure for them to value close emotional bonds (Eagly et al., 2000).

This research provides a new way of understanding casual sex as a response to painful IPV experiences: women respond by becoming more avoidant of closeness. When a relationship partner who should be loving and caring instead is causing pain and trauma (Arriaga et al., 2018), victims will adjust their expectations for how to satisfy their connection needs. Women may be particularly susceptible to feeling undervalued when their partner’s behavior violates relational expectations (Ayduk et al., 1999) and particularly motivated to find another way of connecting romantically with others. Thus, women who experience IPV may turn to casual sex as a means of satisfying connection needs while avoiding the dangers of past violent relationships (Kopetz & Orehek, 2015).

The idea that IPV victimization reinforces attachment avoidant thoughts and feelings is novel and has several implications. This reasoning suggests that attachment avoidance, often considered to be a deficit in relationships, instead may be beneficial for IPV victims. Casual sex provides a rational solution to connecting with others while averting a committed relationship and its associated risks. Thus, attachment avoidance can provide an adaptation to environmental pressures; indeed, attachment theory posits adjustments people make based on their available relationships that can be understood from an evolutionary framework (Szepsenwol & Simpson, 2019).

Another implication is that adult attachment tendencies are not immutable, as revealed in Studies 2 and 3. Repeated instances of being hurt by a relationship partner are salient experiences that may revise a person’s mental model of partners. In the case of IPV victimization, a person learns to be wary of closeness and adopts relatively negative models of partners, which is a specific and reliable feature of an attachment avoidant orientation (Arriaga & Kumashiro, 2019).

More generally, understanding how IPV victims adjust their mental representations of partners may be crucial in devising IPV support interventions. The malleability of attachment-relevant thoughts and feelings opens the possibility of intervention strategies that could specifically target negative mental models of others. Ultimately, it is important to encourage and guide new experiences that can reinforce secure models and promote healthy and satisfying relationships.

### 5.3. Limitations and Directions for Future Research

The current research had several limitations. This research did not examine whether providing women with alternate ways of connecting with others (e.g., community groups) might reduce the desire to seek social connection through risky sexual behavior. Devising and testing alternative ways of attaining social connection without needing to establish an emotional romantic bond could have practical implications for supporting women who have experienced IPV victimization. Also, this research focused on self-identified women; others may have either similar or unique responses to IPV victimization, which remains to be seen in future research.

Finally, Study 3 appropriately relied on experimental methods but was limited by reliance on a hypothetical scenario to assess casual sex. Future research could explore creative ways to test the effect of avoidant (versus secure) thoughts and feelings on the intention to engage in casual sex, while retaining an experimental design that directly tests a causal association.

## 6. Conclusions

Much has been learned about the nefarious effects of experiencing violence from a partner. Some effects are immediate and obvious (e.g., injuries, acute anxiety, and depression). This research examined a more subtle effect: Victims of IPV learn that a relationship partner can cause pain. Victims’ revised way of understanding intimate relationships not only affects their expectations but may also direct them to adopt sexual behaviors with new risks. This research, thus, contributes to our understanding of the psychological factors that unfold from IPV victimization and can inform strategies to alleviate victims’ pain and avert new harms.

## Figures and Tables

**Table 1:** Indirect effects analysis: intimate partner violence predicting risky sexual behavior via attachment avoidance (Study 1; *N* = 312).

	*b* (*SE*)	*p*	95% CI [LL, UL]
**Sexual IPV**			
a-path (Sexual IPV → Attachment Avoidance)	0.22 (0.10)	0.021 *	[0.03, 0.42]
b-path (Attachment Avoidance → Risky Sexual Behavior)	0.23 (0.06)	<0.001 *	[0.11, 0.34]
c-path/Total Effect (Sexual IPV → Risky Sexual Behavior)	0.48 (0.10)	<0.001 *	[0.28, 0.67]
c′-path/Direct Effect (Sexual IPV → Risky Sexual Behavior)	0.43 (0.10)	<0.001 *	[0.24, 0.62]
ab Indirect Effect (Sexual IPV → Risky Sexual Behavior)	0.05 (0.03)		[0.01, 0.10]
**(Severe) Psychological IPV**			
a-path (Severe Psychological IPV → Attachment Avoidance)	0.41 (0.11)	<0.001 *	[0.20, 0.63]
b-path (Attachment Avoidance → Risky Sexual Behavior)	0.19 (0.06)	<0.001 *	[0.08, 0.30]
c-path/Total Effect (Psychological IPV → Risky Sexual Behavior)	0.55 (0.11)	<0.001 *	[0.33, 0.77]
c′-path/Direct Effect (Psychological IPV → Risky Sexual Behavior)	0.47 (0.11)	<0.001 *	[0.25, 0.69]
ab Indirect Effect (Psychological IPV → Risky Sexual Behavior)	0.08 (0.03)		[0.02, 0.15]
**Physical IPV**			
a-path (Physical IPV → Attachment Avoidance)	0.29 (0.10)	0.005 **	[0.09, 0.49]
b-path (Attachment Avoidance → Risky Sexual Behavior)	0.22 (0.06)	<0.001 *	[0.10 0.33]
c-path/Total Effect (Physical IPV → Risky Sexual Behavior)	0.41 (0.10)	<0.001 *	[0.20, 0.61]
c′-path/Direct Effect (Physical IPV → Risky Sexual Behavior)	0.35 (0.10)	<0.001 *	[0.14, 0.55]
ab Indirect Effect (Physical IPV → Risky Sexual Behavior)	0.06 (0.03)		[0.01, 0.13]
**Number of types of IPV**			
a-path (IPV → Attachment Avoidance)	0.11 (0.05)	0.020 *	[0.02, 0.19]
b-path (Attachment Avoidance → Risky Sexual Behavior)	0.22 (0.05)	<0.001 *	[0.11, 0.33]
c-path/Total Effect (IPV → Risky Sexual Behavior)	0.21 (0.05)	<0.001 *	[0.12, 0.30]
c′-path/Direct Effect (IPV → Risky Sexual Behavior)	0.19 (0.04)	<0.001 *	[0.10, 0.28]
ab Indirect Effect (IPV → Risky Sexual Behavior)	0.02 (0.01)		[0.003, 0.05]

Note. * *p* < 0.05, ** *p* < 0.01. All analyses control for attachment anxiety.

## Data Availability

The raw data supporting the conclusions of this article will be made available by the authors on request.
